# Characterization of a Low-Cost Optical Flow Sensor When Using an External Laser as a Direct Illumination Source

**DOI:** 10.3390/s111211856

**Published:** 2011-12-20

**Authors:** Davinia Font, Marcel Tresanchez, Tomàs Pallejà, Mercè Teixidó, Jordi Palacín

**Affiliations:** Department of Computer Science and Industrial Engineering, University of Lleida, Jaume II, 69, Lleida 25001, Spain; E-Mails: dfont@diei.udl.cat (D.F.); mtresanchez@diei.udl.cat (M.T.); tpalleja@diei.udl.cat (T.P.); mteixido@diei.udl.cat (M.T.)

**Keywords:** optical mouse sensor, external laser source, displacement, oscillatory motion

## Abstract

In this paper, a low cost optical flow sensor is combined with an external laser device to measure surface displacements and mechanical oscillations. The measurement system is based on applying coherent light to a diffuser surface and using an optical flow sensor to analyze the reflected and transferred light to estimate the displacement of the surface or the laser spot. This work is focused on the characterization of this measurement system, which can have the optical flow sensor placed at different angles and distances from the diffuser surface. The results have shown that the displacement of the diffuser surface is badly estimated when the optical mouse sensor is placed in front of the diffuser surface (angular orientation >150°) while the highest sensitivity is obtained when the sensor is located behind the diffuser surface and on the axis of the laser source (angular orientation 0°). In this case, the coefficient of determination of the measured displacement, *R*^2^, was very high (>0.99) with a relative error of less than 1.29%. Increasing the distance between the surface and the sensor also increased the sensitivity which increases linearly, *R*^2^ = 0.99. Finally, this measurement setup was proposed to measure very low frequency mechanical oscillations applied to the laser device, up to 0.01 Hz in this work. The results have shown that increasing the distance between the surface and the optical flow sensor also increases the sensitivity and the measurement range.

## 1. Introduction

The main part of an optical computer mouse is currently an optical flow sensor that estimates the relative displacement of the surface under the mouse by tracking the micro-shadows of the surface [1,2]. The optical flow sensor is a motion sensor that measures linear displacements very well (with a very high coefficient of determination, *R*^2^ = 0.99 [3]) although it is very sensitive to height changes [1,4], very dependent on the measurement surface [5], and sensitive to the trajectory [3,4]. Despite these limitations, the versatility and low cost of this mass-produced industrial sensor has fostered the development of a wide range of specific applications. In [1], the optical sensor was used to estimate the viscoelastic deformation of polyethylene. In [2,6], it was used to estimate mobile robot displacement. In [7], four optical mouse sensors were used as sheet position sensors in a printer paper path and in [4], multiple optical sensors mounted on a rigid body were used for trajectory measurement. In [8], the optical mouse sensor was used to estimate the position changes caused by a tethered subject (a parasitic fly *Ormia ochracea*) located on the spherical surface of a ping-pong ball which was kept floating over the optical sensor with an air pressure tube. In [9], a minimally invasive surgical instrument (MIS) was designed as a gimbal mechanism to allow small movements in the incision point due to large human movements of the tip of the instrument. In [10], the internal sensor registers were used to analyze a rotary white surface and locate a thin black radial reference line. In [11], two external laser devices were used with the optical flow sensor to estimate the position of a mobile robot on slippery terrains. In [12], the optical sensor with specific lenses was used in an Unmanned Aerial Vehicle (UAV) providing information about its current velocity using the texture of the surface under the UAV.

There are also specific applications based on the image capabilities of the optical flow sensor. In [13], the optical sensor was used as a counterfeit coin detector by analyzing the partial images of a 2-euro coin. In [14], a similar proposal was used to develop an absolute rotary encoder by reading a binary code in a rotary surface.

### 1.1. Related Work

The proposal of combining a laser with a camera in order to develop different measurement systems has many applications. In [15], this measurement setup was applied to measure the waveform produced by the arterial dorsum manus vibration for patients with Parkinson’s disease. In [16], this measurement setup was used as a biospeckle technique to estimate the quality of apples. The measure was based on the analysis of variations of laser light applied on apples’ surface which is related to their values of firmness, soluble solids content, titratable acidity, and starch content. In [17], this measurement setup was used as a non-destructive weld quality inspection to detect weld defects on surfaces improving the quality of the components.

In the case of low-cost optical flow sensors the original assembly illuminates asymmetrically the surface under the sensor and generates micro-shadows in one predominant direction and this asymmetrical illumination was reported as a possible cause of different measurement problems [3,4]. In [11,18] the use of an external illumination system was proposed to avoid the problems originated by the original asymmetrical illumination. In [19], the proposal was to use an external laser device to illuminate the surface under the optical flow sensor and a set of different measurement experiments were proposed and analyzed to validate this proposal: monitoring the paint drying process on a coin, measuring the variation in the diameter of a radial artery due to the heart beat, estimating the angular displacement during the rotation of a steel sphere, and estimating the translational movement of the middle point of a wooden bar due to the load applied. One of the conclusions obtained in [19] was that an external laser device and a reflective surface can be used to enlarge the operational distance between the diffuser surface and the mouse sensor up to 300 mm. This conclusion opened a new range of applications and the aim of this work is to go further in this direction.

### 1.2. New Contributions

The main goal of this work was to explore the new possibilities that arise when using an external laser device with a low-cost optical flow sensor. These new contributions were the definition of a measurement setup based on an auxiliary diffuser surface that can be used in two different ways: measuring the displacement of the surface, or measuring the displacement of the laser spot over the surface. In both cases the measurement system was characterized. The main advantages of this measurement setup were: (1) the sensitivity of the measurement of the displacement can be modified by adjusting the distance between the surface and the optical flow sensor. (2) the system can be used to measure very low mechanical oscillations applied to a laser device (measuring the projected laser spot displacement).

## 2. Materials and Methods

The material used in this work was an optical flow sensor, a laser device, and a diffuser plastic material. The laser spot is pointed to the diffuser material and is microscopic structure was refracted and transmitted and captured in the image received and analyzed by the optical flow sensor. Therefore, with such configuration the optical flow sensor should be able to detect small displacements of the diffuser surface and also relative displacements of the laser spot.

### 2.1. Optical Flow Sensor

**Figure 1.**
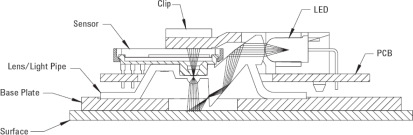
Cross section view of assembly components for the optical sensor ADNS-3080 (courtesy of Avago).

The optical flow sensor selected for this work is the ADNS-3080 [20] (Figure 1), manufactured by Avago Technologies (San Jose, CA, USA), a common optical flow sensor that includes an internal low-resolution camera and a digital signal processor (DSP) programmed to estimate the relative displacement of the micro shadows of the images acquired [21]. This family of optical sensors uses standard and versatile Complementary Metal-Oxide-Semiconductor (CMOS) technology that integrates the camera and the DSP into the same chip. The sensor computes the optical flow by performing a comparative analysis of the sequence of images acquired of a flat surface in front of the sensor in order to estimate the motion of the surface; a detailed description of the algorithms used can be found in [22]. The motion can be measured at different resolutions such as 800 counts (or incremental pulses) per inch (CPI). Under this specific configuration, the optical flow sensor is internally calibrated to measure one pulse when a plain surface at 2.4 mm in front of the sensor is translated 31.75 μm and any change in the original measurement setup proposed by the manufacturer (such as a change in the distance between the sensor and the surface) will require the development of a specialized calibration procedure to convert the counts measured by the sensor in an estimate of the physical magnitude under measurement.

The original design of an optical mouse based on this optical sensor combines different parts (Figure 1): an optical flow sensor, an external light emitting diode (LED) to illuminate the surface in front of the sensor, and a small plastic structure that includes two convex lenses to focus the light of the LED and the image acquired by the optical sensor. The combination of all these parts was optimized to acquire focused images of a surface at a very short distance (from 2.3 to 2.5 mm), although this range can be increased by replacing the original lens provided. The optical flow sensor has an internal closed loop to control the intensity of the LED by using a dedicated pulse width modulation (PWM) output.

The ADNS-3080 includes an internal imaging device with a sensitive array of 30 × 30 gray intensity pixels. This optical sensor operates at very high frame rates and has a very fast internal shutter. It was originally designed to acquire images of a flat surface (approximately 1.82 × 1.82 mm) at a very short distance (2.4 mm) to estimate displacement within images.

The ADNS-3080 has a standard Serial Peripheral Interface (SPI) bus to read/write the internal registers and control the common actions of the sensor. In normal operation, the displacement of the flat surface under the sensor is obtained by reading the Delta_Y and Delta_X registers that return the relative movement measured. According to the manufacturer’s specifications, the optical sensor can acquire and process images at a very fast speed: from 2,000 to 6,469 frames per second, due to the low size of the images acquired (30 × 30 pixels). Finally, the optical sensor has a special motion burst access mode that requires only 107 μs (when operating with the SPI bus at full speed) to read all the internal registers that define the motion measured.

### 2.2. Laser Device

**Figure 2.**
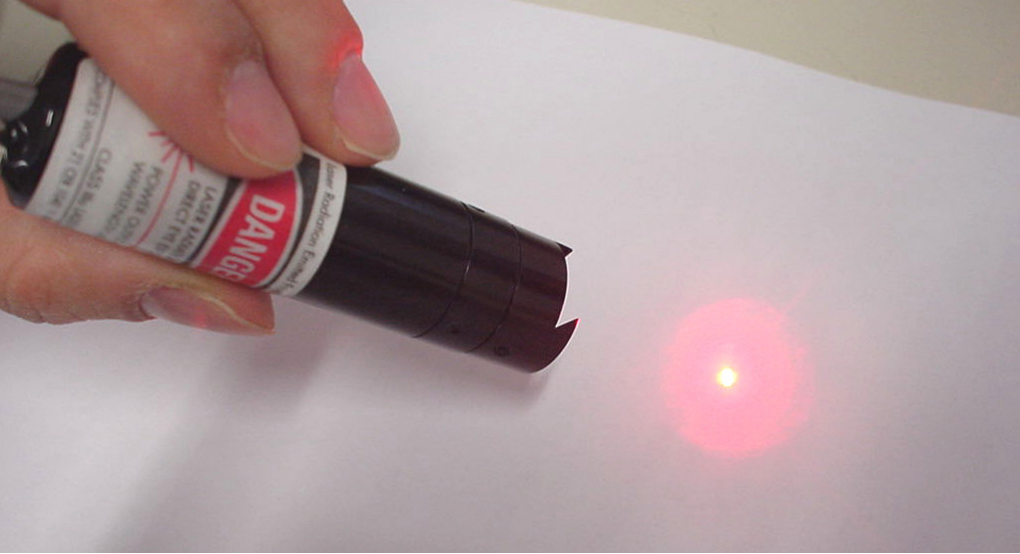
Image of the Lasiris Mini 660 and its spot.

The laser device used in this work is the Lasiris Mini 660 manufactured by Coherent Inc (Santa Clara, CA, USA); a class IIIa solid state laser source with a power output below 5 mW, diode power of 35 mW, and a wavelength of 660 nm (Figure 2).

### 2.3. Diffuser Material

**Figure 3.**
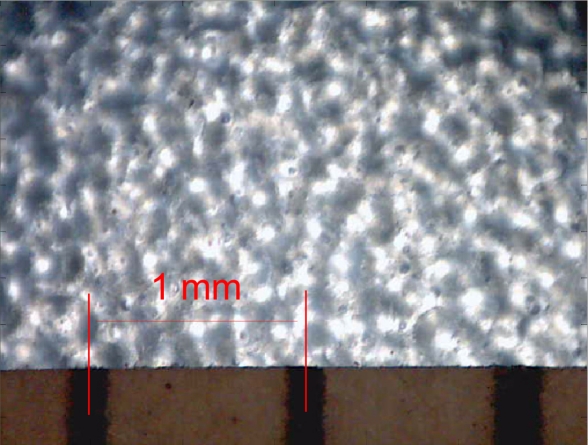
Image of the diffuser paper obtained with an optical microscope: backlight illuminated.

The diffuser material used in this work was a common industrial polypropylene plastic paper: thickness 0.62 mm, roughness 0.961 μm, density 550 gr/m^2^, and index of transmission 0.77 at 660 nm. Figure 3 shows a microscopic image of the diffuser paper with a white backlight illumination. This image reveals a structure that generates a large number of intensity peaks and valleys that very closely resemble the micro-shadow features [21] required by an optical flow sensors to estimate surface motion. Therefore, the structure of this thin material is ideal to perform optical flow measurements.

## 3. Measuring Surface Displacement

This section analyzes the performances of a measurement setup designed to estimate vibration or small displacements of the diffuser surface. In this measurement setup, the laser device and optical flow sensor were fixed while the diffuser surface was free but attached by some means to the place or object under measurement. The effect of the relative angular orientation and distance of the optical sensor placement was analyzed.

### 3.1. Effect of the Relative Angular Orientation of the Sensor

**Figure 4.**
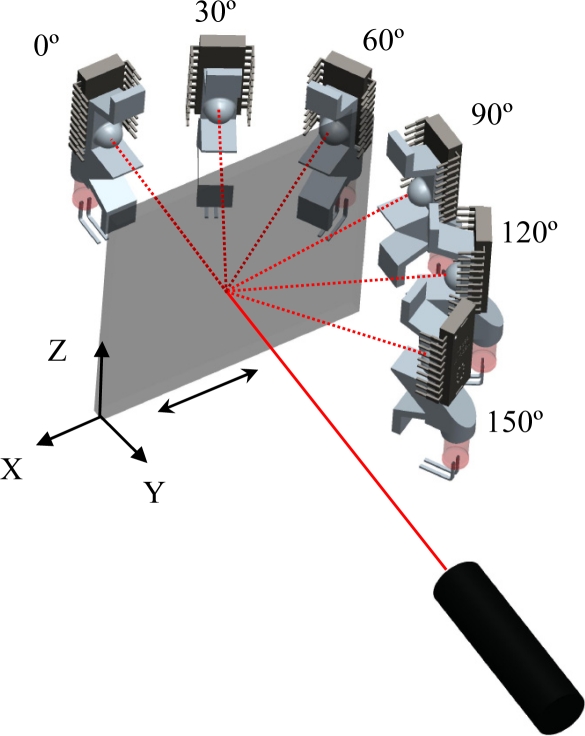
Configuration of the experimental setup used in the first and second experiment.

A first experiment was carried out to evaluate the effect of the relative angular orientation of the optical sensor on the measurement of surface displacement. Figure 4 depicts the configuration of the measurement setup used in this experiment, consisting of the external laser device, the optical flow sensor, and the diffuser surface. The laser was fixed 300 mm in front of the diffuser surface and the optical flow sensor was placed at a radial distance of 150 mm from the diffuser surface at different relative angular orientations: from 0° (laser axis, behind the surface) to 180° (laser axis, in front of the surface) in steps of 30°.

**Figure 5.**
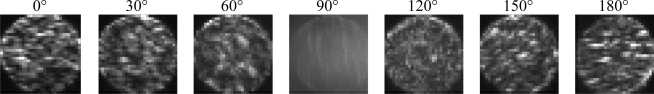
Image acquisition for different angular position of the optical sensor.

Figure 5 shows the images acquired by the optical sensor at the different angular orientations considered. The optical sensor was oriented to the reflected (Figure 5, 180°) or transmitted (Figure 5, 0°) laser beam and the common characteristics of the images acquired in this experiment are that they have an illuminated central circumference showing the inner transmitted or reflected microscopic structure of the diffuser surface. This circular shape was caused by the convex plastic lens used by the optical sensor and by the non-diffused illumination used. Additionally, we have observed that that a change in the size of the laser spot does not affect the radius of this circumference, and this circular shape remains static for small displacements of the diffuser surface while the inner image changes. Therefore, any motion applied to the diffuser surface only affects the inner texture of the image that is displaced proportionally and this image displacement can be measured directly by the optical sensor as it computes the optical flow of the images. The application of an optical flow algorithm to one image requires the existence of areas with different intensities (also called features) to correctly detect any motion. Therefore, Figure 5 shows that when the optical sensor was placed at 90° (perpendicular to the diffuser surface) the intensity of the transmitted or reflected light reaching the sensor was very homogeneous (without features) and, as a consequence, the optical flow motion measurement will fail.

A second experiment was carried out to calibrate the displacement of the diffuser surface for the different angular orientations considered in Figure 4. In this case the diffuser paper was displaced 10, 20, 30, 40, and 50 mm along the X-axis, each displacement was repeated five times in each relative angular orientation. During this experiment the optical flow sensor was configured to measure motion with a resolution of 800 counts per inch (CPI).

**Figure 6.**
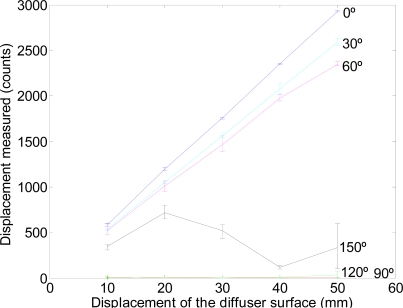
Relationship between the counts measured and the displacement of the diffuser surface for different angular orientations.

Figure 6 shows the average, maximum and minimum counts results obtained. The agreement between a linear model and the data was very good (*R*^2^ > 0.99) when the optical flow sensor was placed at angular orientations from 0° to 60°; the highest linearity and sensitivity was obtained when the optical flow sensor was located at 0° (see Figure 4). Figure 6 shows that the displacement of the diffuser surface is not correctly measured at angular orientations from 90° to 180° probably because the reflected image appears blurred and because the roughness of the surface does not produce reflections in a predominant direction that could be measured by the internal optical flow algorithms of the sensor.

Equation (1) shows the linear regression computed for the three best cases where *α* is the angular orientation, *d* is the displacement of the diffuser surface along the X-axis in millimeters, *c̅* is the estimate of the counts measured by the system, and *R^2^* the coefficient of determination of the linear fitting. The maximum sensitivity [Equation (1a)] was 58.36 counts per millimeter (CPM) at an angular orientation of 0°.

(1) $$\begin{array}{l} a)\;\;\;\;\;\alpha =0{}^{\circ},\;\;\;\;\;\;\bar{c}=58.36\cdot d+12.60,\;\;\;\;\;\;R^{2}=0.99\\ b)\;\;\;\;\;\alpha =30{}^{\circ},\;\;\;\;\;\bar{c}=51,52\cdot d+16.27,\;\;\;\;\;\;R^{2}=1.00\\ c)\;\;\;\;\;\;\alpha =60{}^{\circ},\;\;\;\;\;\bar{c}=46.08\cdot d+81.80,\;\;\;\;\;\;R^{2}=0.99 \end{array}$$

### 3.2. Effect of the Distance between the Surface and the Sensor

**Figure 7.**
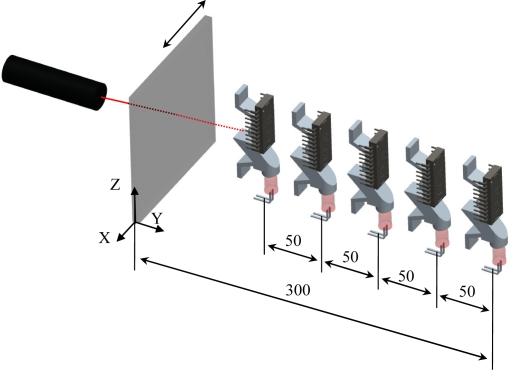
Configuration of the experimental setup used on the surface-sensor’s distance effect.

The third experiment was carried out to evaluate the influence of the distance between the diffuser surface and the sensor on the measurement performed by the optical sensor. Figure 7 shows a representation of the measurement setup used in this experiment with the optical flow sensor fixed at angular orientation 0° (laser axis, behind the surface). This angular orientation was selected because in the previous experiment it has the highest motion sensitivity. In this experiment, the diffuser surface was displaced 50 mm along the X-axis while the distance from the diffuser surface and the optical sensor were 100, 150, 200, 250 and 300 mm, in agreement with the distance of 300 mm used in [19] while performing a similar measurement. The measurements were repeated five times at each distance.

**Figure 8.**
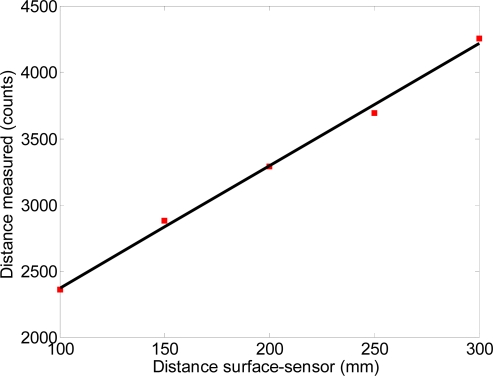
Counts measured by the optical sensor when the diffuser surface was displaced 50 mm (red points) and linear regression (solid dark line).

Figure 8 shows the average counts measured by the optical sensor relative to the distance between the surface and the sensor when the diffuser surface was displaced 50 mm. These results show that the counts measured increased as the distance also increased, with a high linearity in this relationship, *R*^2^ > 0.99. Therefore, the sensitivity of this combined measurement system can be modified by selecting the appropriate distance between the diffuser surface and the optical sensor. This feature is specially interesting as it enables the selection of the highest dynamic range in a particular displacement measurement.

Finally, the relative error in the counts measured relative to the counts estimate obtained by using the linear regression decreased as the distance between the diffuser surface and the optical sensor increased; the maximum relative error was 1.29% at the shortest distance, 100 mm. These results agree with the results obtained in [18] and [19] where a similar setup was used to measure small displacements placing an optical flow sensor at 300 mm of a reflecting surface.

## 4. Measuring Laser Spot Displacement

**Figure 9.**
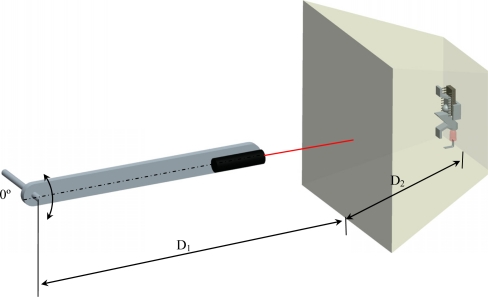
Configuration of the experimental setup used to measure spot displacement.

In the previous section, the laser and the optical flow sensor were fixed and the diffuser surface was displaced. In this section, the proposed measurement setup (see Figure 9) has the diffuser surface and the optical flow sensor fixed (defining a compact optical measurement system) while the displacement is applied directly to external and free laser. Figure 9 shows a fixed box containing the diffuser surface and the optical sensor inside; the laser was attached externally to a pivoting arm to displace the spot along the diffuser surface. The distance between the pivoting point of the arm and the diffuser surface (Figure 9, D_1_) was 443 mm, the distance between the laser and the diffuser surface was 100 mm, and the distance between the diffuser surface and the optical flow sensor (Figure 9, D_2_) was changed from 100 to 400 mm in 100 mm steps.

**Figure 10.**
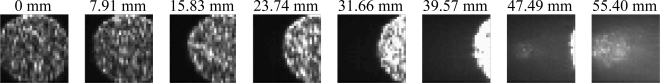
Image acquired by the optical flow sensor for different vertical displacements of the laser spot along the diffuser surface.

Figure 10 shows a sequence of images that represent the image viewed by the optical flow sensor during half of a period of an oscillatory motion applied to the arm that holds the laser when the distance between the sensor and the diffuser surface was 200 mm. As stated previously, the image viewed is not the laser spot, but rather the transmitted light that reaches the optical flow sensor through the original convex lens that adopts this particular circular shape. Figure 10 shows the effect of the displacement of the spot in millimeters along the diffuser surface. Figure 10, 0 mm case, shows the image acquired by the optical flow sensor when the spot of the laser was at the center of the area covered by the optical sensor. The optical flow sensor was able to measure displacement while part of the illuminated central circumference was inside the image acquired and measurements failed when the circumference disappears from the optical sensor’s field of vision.

The following experiment was planned to evaluate the maximum displacement of the laser spot that can be measured with the proposed measurement system. In this case, a sinusoidal oscillation of variable amplitude was applied to the arm that holds the laser. This experiment was repeated for different distances between the diffuser surface and the inner optical flow sensor: 100, 200, 300 and 400 mm, the maximum range allowed by our measurement setup.

**Figure 11.**
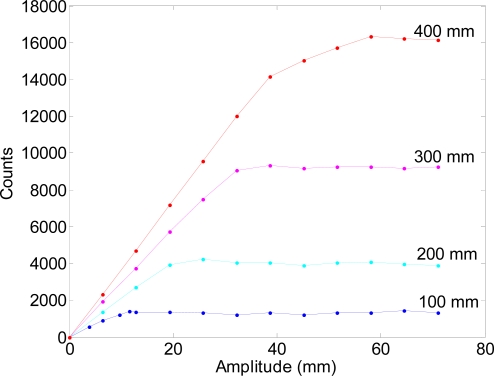
Counts measured by the optical mouse sensor relative to the amplitude of the sinusoidal oscillation projected by the laser onto the diffuser surface for different inner distances between the diffuser surface and the optical mouse: 100, 200, 300 and 400 mm.

Figure 11 shows the counts measured by the optical sensor relative to the amplitude of the spot displacement over the diffuser surface. The counts measured by the optical flow sensor saturated when the illuminated central circumference disappeared from the optical sensor’s field of vision because then no features of the surface could be tracked by the internal optical flow algorithm. Figure 11 shows that the range of the amplitude measured increased as also increase the distance between the diffuser surface and the optical flow sensor. The linear part of the relationship shown in Figure 11 was modelled in Equation (2) where *w* is the distance in millimeters between the optical flow sensor and the diffuser surface, *p̅* is the estimate of the amplitude of the counts measured by the system, *A* the maximum amplitude in millimeters of the sinusoidal oscillation measured, and *R^2^* the coefficient of determination or the linear fitting (expressed as *p̅* = *m* · *A* + *b* where *m* is the sensitivity of the measurement setup).

(2) $$\begin{array}{l} a)\;\;\;\;w=100,\;\;\;\;\;\bar{p}=110\cdot A+100,\;\;\;\;\;\;R^{2}=0.97\\ b)\;\;\;\;\;w=200,\;\;\;\;\bar{p}=200\cdot A+26,\;\;\;\;\;\;\;\;R^{2}=0.99\\ c)\;\;\;\;\;w=300,\;\;\;\;\;\bar{p}=280\cdot A+92,\;\;\;\;\;\;\;R^{2}=0.99\\ d)\;\;\;\;\;w=400,\;\;\;\;\;\bar{p}=370\cdot A-18,\;\;\;\;\;\;\;\;R^{2}=0.99 \end{array}$$

The maximum sensitivity was 370 CPM [Equation (2d)] when the sensor was 400 mm behind the diffuser surface. Additionally, the values of the sensitivity have a linear relationship (*R*^2^ = 0.99) with the distance from the optical flow sensor and the diffuser surface, *w*:

(3) m=0.86⋅w+25

Therefore, Equation (3) can be used to estimate the sensitivity (expressed in CPM) of the proposed measurement system at a design stage. Additionally, there is apparently no relationship between the constant term, *b*, and the distance *w*. Finally, there is also a relationship between the maximum amplitude of the oscillation measured before saturation and the distance between the diffuser surface and the optical flow sensor:

(4) $$\bar{A}=49.65\cdot w-4661$$

where *Ā* is the estimate maximum amplitude of the oscillation in millimeters that the system can measure before saturation. This relationship can be expressed alternatively as:

(5) $$\bar{w}=0.019\cdot A-99.82$$

where *A* is the maximum amplitude of the oscillation to measure (in millimeters), and *w̅*, the estimate of the minimum distance between the diffuser surface and the optical flow sensor to cover the selected amplitude of the oscillation of the laser spot. In both cases the coefficient of determination was 0.96 with a very high agreement with the linear model.

The last experiment performed in this work was carried out to evaluate if the proposed system can measure the amplitude of the oscillation at very low frequencies. In this case the measurement setup was the same as described before and the distance between the optical sensor and the diffuser surface was 300 mm. The oscillatory motion of the arm that holds the laser was generated continuously by controlling the speed of different DC geared motors attached to the arm. The range of frequencies analyzed was from 0.01 to 1 Hz that are the frequency limits of the mechanical system used in this experiment.

**Figure 12.**
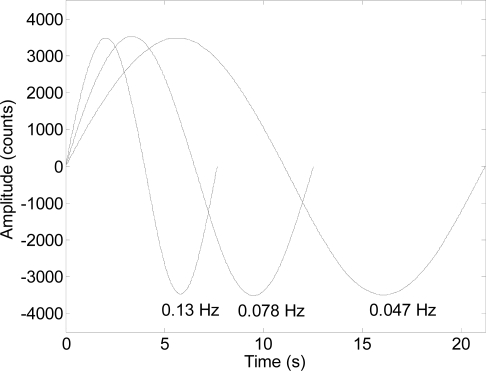
Evolution of the counts registered during the oscillatory motion of the laser spot obtained for three different frequencies: 0.047, 0.078, and 0.130 Hz.

Figure 12 shows the evolution of the register of the raw data counts obtained with the proposed measurement system corresponding to three mechanical oscillations with fixed amplitude and frequencies of 0.047, 0.078 and 0.130 Hz; the sampling time was 5.44 ms. The amplitude was expressed in counts but can be converted into millimeters simply by applying a conversion factor.

**Figure 13.**
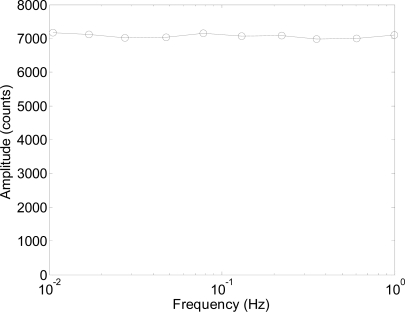
Peak to peak oscillation amplitude obtained when registering different sinusoidal oscillations of fixed amplitude.

The results in Figure 12 show that the sinusoidal shape of the oscillation was perfectly registered in all cases. Note that the peak to peak amplitude of the oscillation was 7,000 counts and this value represents more than 200 times the resolution of the internal image acquisition device of the optical flow sensor (30 × 30 pixels) and this is because the optical flow sensor tracks the displacement of the microscopic features projected on the image. The only drawback of this measurement setup is that a linear offset can appear in the raw data if the zero position of the laser spot projected onto the diffuser surface is not in the center of the image acquired by the optical flow sensor. This effect is caused by the low-cost convex lens originally provided with the optical flow sensor. Finally, Figure 13 shows the peak-to-peak amplitude of the laser spot oscillation expressed in counts for different sinusoidal frequencies. The peak-to-peak amplitudes obtained were very similar for all frequencies tested, confirming that this system can be used to measure oscillations with very low frequencies. The lowest operational frequency range defined for micro-electro mechanical accelerometers is usually 1 Hz [23–29] so this proposal opens a new range of applications that require the measurement of low frequency oscillations with a low-cost measurement setup.

## 5. Conclusions

This work is focused on the experimental characterization of a low-cost measurement setup based on the use of a common optical flow sensor and an external laser as the illumination source. The coherent light generated by the external laser device used was applied to a diffuser surface and the transmitted and reflected light was projected with the embedded convex lens into the image acquisition device of the optical sensor and used to measure the motion of the surface and the displacement of the laser spot over the surface. The characterization of this new measurement system was performed by placing the optical sensor at different angular orientations and distances from the diffuser surface.

The experimental results obtained show that the distance from the diffuser surface and the optical flow sensor can be increased greatly when using an external laser source. The results show that image acquired with the internal image acquisition device has a typical circular illuminated shape that contains the features detected by the optical flow algorithm. Under such conditions, the original recommended operational distance of 2.4 mm can be increased considerably. In a previous work the distance tested was 300 mm and in this work a range from 100 to 400 mm was explored. Two different measurement strategies relying on this effect were proposed. The first approach consisted of measuring the motion applied to the diffuser surface and the second consisted of measuring the motion applied to the laser spot that was projected onto the diffuser surface. In both cases, the optical flow sensor was able to detect motion while the image acquired by the optical sensor contained features (microscopic image differences) that can be tracked by the optical flow algorithm implemented in the sensor. The results show that the highest motion sensitivity was obtained when the optical flow sensor was located behind the diffuser surface and on the axis of the laser source.

The conclusion of the experiments performed was that a measurement setup consisting of a diffuser paper in front of an optical flow sensor can be used to measure the mechanical oscillation of a laser spot. The mechanical vibration or oscillation applied to the laser device can be measured with a sensitivity that depends on the distance between the diffuser paper and the optical flow sensor. The resolution achieved with this measurement setup was more than 200 times higher than the pixel resolution of the internal camera of the optical flow sensor.
